# Valuing biomarker diagnostics for dementia care: enhancing the reflection of patients, their care-givers and members of the wider public

**DOI:** 10.1007/s11019-018-09883-2

**Published:** 2019-01-24

**Authors:** Simone van der Burg, Floris H. B. M. Schreuder, Catharina J. M. Klijn, Marcel M. Verbeek

**Affiliations:** 1 Wageningen University & Research, Wageningen Economic Research, Hollandseweg 1, 6706 KN Wageningen, The Netherlands; 2 Department of Neurology, Donders Institute for Brain, Cognition and Behaviour, Radboud University Medical Center, PO Box 9101, 6500 HB Nijmegen, The Netherlands; 3 Department of Laboratory Medicine, Radboud University Medical Centre, PO Box 9101, 6500 HB Nijmegen, The Netherlands

**Keywords:** Early diagnostics, Dementia, Ethics, Stakeholder discussion, Care, Cure

## Abstract

What is the value of an early (presymptomatic) diagnosis of dementia in the absence of effective treatment? There has been a lively scholarly debate over this question, but until now (future) patients have not played a large role in it. Our study supplements biomedical research into innovative diagnostics with an exlporation of its meanings and values according to (future) patients. Based on seven focusgroups with (future) patients and their care-givers, we conclude that stakeholders evaluate early diagnostics with respect to whether and how they expect it to empower their capacity to (self-) care. They value it, for instance, with respect to whether it (a) explains experienced complaints, (b) allows to start a process of psychological acceptance and social adaptation to the expected degeneration, (c) contributes to dealing with anxieties (with respect to inheritable versions of dementia), (d) informs adequately about when to start preparing for the end of life, (d) informs the planning of a request for euthanasia, or (e) allows society to deal with a growing amount of dementia patients. Our study suggests that information about disease is considered ‘harmful’ or ‘premature’ when recipients feel unable to act on that information in their (self-) care. The results of this research offers input to further ethical research. It invites to adopt a care perspective in evaluation and to seek ways to prevent the ‘harm’ that such diagnostic methods can bring about.

## Introduction

The rapidly aging population and the rising prevalence of dementia in society is causing many societal concerns, to which diverse academic fields seek an appropriate response (Winblad et al. 2016). As the gradual cognitive degeneration of patients with dementia has an enormous impact on the lives of these patients and their relatives, as well as on society, scientists have engaged in various investigations in order to find an appropriate response to it. Neurochemical research into biomarkers that indicate early stage dementia—or which are able to predict dementia pre-symptomatically—is one example of such scientific investigations. Such neurochemical biomarkers, usually quantified in cerebrospinal fluid (CSF), support to define a diagnosis that is expected to differentiate between different types of dementia in patients during life (as opposed to post mortem examination) and it may offer a useful quantitative scale to monitor the effects of experimental pharmacological treatment during a clinical trial. Yet, early (presymptomatic) diagnostics of Alzheimer and other types of dementia also raises ethical debate; for what is the value of having a (predictive) diagnosis, especially when it is unsure whether effective treatment will become available? What are the the harms and benefits for the lives of patients when this diagnosis is communicated to patients?

There is debate about this question in the social sciences and in ethics. Many scholars in these fields regard dementia biomarker research with reserve (Jongsma and Sand 2017; Cuijpers and Van Lente 2015; Innes and Manthorpe 2013; Peine et al. 2015; Boenink et al. 2011; Van der Laan and Olthuis 2013). Some criticize it for offering reductionist ideas about what dementia is and ‘how we might try to respond to or look for a solution’ to it (Innes and Manthorpe 2013). Others state that biomarker research presupposes that dementia is a disease with a biochemical source, while it could just as well be understood quite differently as a set of functional problems or part of normal aging (Moser 2008, 2011). It matters, according to these scholars, how dementia is understood, as these understandings invite different kinds of responses. Looking for a cure (a so-called ‘technological fix’) would make sense if dementia is understood as a disease with a biochemical source. However, some argue that dementia also needs to be approached in a broader way as history points out that the hope to find a cure often fails to materialize, or materializes in a sub-optimal manner, such as is the case with promises to provide a ‘cure for cancer’ (Jongsma and Sand 2017). The focus on finding a cure for dementia should therefore not eclipse attention for more *low tech* ‘caring’ responses that dementia patients should be able to fall back on—and which represent, according to some, a much better strategy to deal with dementia (Cuijpers and Van Lente 2015).

This scholarly debate has been taking place over some time now. But it is not very clear yet, how (future) patients would value having a (predictive) test. Interest in the perspectives of patients on biomedical research is growing in the past years (Van der Scheer et al. 2014). It has been promoted for different reasons: such as, for the democratization of science (Nowotny 2003), to improve alignment of biomedical innovation with the wishes of patients as the consumers of health care services (Boote et al. 2006), to make the biomedical research agenda responsive to the needs of patients (Abma and Broerse 2010), or to improve the quality of biomedical research by means of a coupling of patient’s experiential knowledge with the disease to the biomedical knowledge of scientists (Caron-Flinterman et al. 2005). In the domain of research into Alzheimer’s diagnostics, not a lot of research into patient perspectives have been done until now. While several authors have recommended organizing stakeholder engagement dialogues to anticipate the socio-cultural meanings attached to early (presymptomatic) Alzheimer’s diagnostics and assess its value for patients, there have until now been little attempts to actually do so (Peine et al. 2015; Boenink et al. 2011). As interesting exception, a recent study compared care and cure-oriented meanings attached to Alzheimer used in health technology assessment with the interpretations provided by patients and their care-givers in Alzheimer cafés (Cuijpers and Van Lente 2015). This work was, however, conceptual and did not actually offer an assessment of the value of biomarker diagnostics for the future lives of patients.

In our research, we aimed to fill this gap. Our goal was to provide insight into what the meaning is of biomarker diagnostics in the lives of (future) patients, and whether and how they value the various possible roles that it can play in their lives. This is an empirical exploration. Yet, we think it can offer valuable input to ethical reflection. As ethics of the ‘good life’ enquires into what the good life is, and what it means to live well, it seems appropriate to explore whether and how people with a disease such as dementia, value an innovative method that allows to set their diagnosis early and if it strengthens or weakens their capacity to live a good life. This is important empirical input to an ethical assessment of the envisioned diagnostic method.

## Method

The study we describe in this article was part of a larger multi-center research project investigating biomarkers in the cerebrospinal fluid CSF indicative of Cerebral Amyloid Angiopathy (CAA). The gradual increase of these biomarkers is considered explanatory for dementia complaints. CAA also increases the risk for intracerebral hemorrhage (ICH), which can lead to death or—depending on the location where it strikes- to mild to severe disability. Patients who survive their ICH are at increased risk for dementia, with an incidence of 28% up to 4 years after ICH (Moulin et al. 2016). Moreover, in CAA patients without ICH, the risk of dementia is very high; up to 73% after 5 years of follow-up (Xiong et al. 2017).

The multi-center research project aimed to investigate the CAA biomarkers. But the project also contained a workpackage on ethics and patient involvement, which was carried out at the same time. In this workpackage we explored the concepts and values that play a role in the viewpoints of end users, defined as (future) patients and their care-givers. We wanted to find out what the meaning of (predictive) diagnostic knowledge is for their lives, and what personal and societal values they ascribe to it. This work package started out as ‘ethical parallel research’, which supplements research into a new technology with an (empirical) ethical investigation that aims to explore and evaluate its impacts on the lives of future users, such as patients (Van der Burg 2009; Van Gorp and van der Molen 2011; Van der Burg and Swierstra 2013; Flipse et al. 2013). The purpose of ‘parallel’ research is that it aims to engage end-users in scientific (technological) research, anticipate impacts of research and innovative technology on human (social) live, and enhance reflection about its value among the various stakeholders. In this sense it fits with calls for responsible research and innovation (Burget et al. 2016; Guston et al. 2014; von Schomberg 2011). At the end of this research, however, the ethical part was no longer carried out in parallel, but it became integrated in the project: as the results lead to lively discussions with physicians and chemical neurologists who took part in the project, which helped to shape the discussion-part of this article, we eventually decided to co-author this article.

## Design

As our goal was to explore what meanings and values (future) patients and care-givers ascribe to (early) CAA diagnostics, we needed to enhance their reflection on something that they probably did not think about before. Early CAA diagnostics is still a topic for research, so chances are small that participants in our research encountered it in their daily lives and thought about it a lot prior to our research. We therefore chose to engage participants in focusgroups, as this is a common way to generate reflection and discussion about a wide variety of topics, and we used cards to be able to give input to their reflective exchange.

Cards are regularly used in qualitative research, and are appreciated as a way to engage patients (even illiterate ones) in a discussion, especially when they are reluctant to speak or are afraid they may have nothing to say about the subject (Kitzinger 1994). Cards provide input to reflection and invite a response, even when topics are experienced as shameful or sensitive (Chang et al. 2005; Sutton et al. 2011). Cards are also used in a different domain; namely, to stimulate reflection and debate about topics that people do not usually think about, such as when members of the public are asked to reflect on the design of innovative artefacts (Sealea et al. 2002), or when laymen in science are demanded to explore or assess the value of scientific or technological futures (Davies and Macnaghten 2010; Felt et al. 2014; https://playdecide.eu).

As early (presymptomatic) test of CAA is a method that the people in our focusgroups likely have little experience with, we chose to use a card-game method for our focusgroups (Boenink et al. 2011; Felt et al. 2014). This card game enhances the imagination of participants in a structured and focused way, about the possible impacts of the CAA detection method on their own lives and on society. This is done by means of differentsets of cards, which each provide input to a round of the conversation. Here we chose to do three rounds of conversation and made three sets of cards. While the card method has sometimes four stages (and four sets of cards) we wanted to limit the needed time-investment of the participants.

1. *First round*. The first set of cards present four possible future uses of biomarker information for CAA and participants were invited to react intuitively.
2. *Second round.* The second set of cards present personal values which invite participants to articulate and reflect on the these future uses for their own personal lives.
3. *Third round.* The third set of cards offer statements or opinions related to the value of various future uses for society.

In each of the conversation rounds, participants received cards with different input for their reflection. But the cards function only as a reflection and discussion starter; they do in no way determine the course or content of the conversation. What is specific about our card game, and it has this in common with the IMAGINE card game by Felt et al. (2014), is that the choice of cards is kept at the level of the individual. Each participant gets his or her set of cards and chooses the cards that are relevant to him or her, and shapes what he or she wants to say in the focusgroup in agreement or in contrast with these cards. The cards will therefore give input to individual reflection, and help to form thoughts about a new subject, but participants in the focusgroup are subsequently free to do with the cards as they please: so, they can choose topics from the cards, or ignore them, or add topics.

To make the cards effective reflection and discussion enhancers, we gave them a content specific to this project. Based on an exploration of the literature describing the societal value of (early) diagnostics of dementia, we developed an interview guide to conduct interviews (live and by phone) with project members and five patient-representatives: two members of the HCHWA-D patient organization who have an inheritable version of CAA which leads to early onset dementia or ICH, two from Alzheimer NL, and one patient who suffered a stroke from the Heart Foundation. Based on the interviews we identified four possible future uses of (presymptomatic) CAA detection in brain fluid (see Box 1), which we described in the first set of cards.

**Box 1:** First set of cards—possible future uses

– For diagnostics: helping to set the diagnosis after patients present themselves to a medical doctor with complaints
– For population screening: detecting an early stage of CAA development in pre-symptomatic healthy middle aged adults (say, 45 +), which implies a risk to develop dementia complaints later in life
– For research: allowing to monitor the effect of experimental medicine on the associated biomarkers in CSF
– For monitoring: keep check of the development of the disease in patients in order to know when they can expect their complaints to worsen
– Empty card

In addition to future uses, we described a list of personal values that came forwards in the interviews (see Box 2) and a list of societal dilemma’s (Box 3), based on the literature and the interviews. In all sets of cards we added empty cards to allow participants to add additional uses, personal values or societal issues that participants in the focusgroups thought were missing on the cards.

**Box 2:** Second set of cards—personal values

Knowledge	Care	Safety	Acceptance
Control	Life	Medicalization	Open future
Autonomy	Hope	Relationships	Empty card

**Box 3:** Societal issues

• Costs should not play a role in the selection of a diagnostic method for CAA
• Society should primarily invest in the provision of good care for people with dementia, not in research into medication
• When an instrument will be available to measure CAA in healthy people, then the results should be shared with family, colleagues, insurance and employers
• It is a good idea to have a CAA-screening program for healthy people, for this will foster research into the natural development of the disease or into medication that serves the health of future patients
• Empty card

For the focusgroups with people with dementia we simplified the card-game method, after consulting care-givers and social scientists with experience in focusgroups with people with dementia. They told us to limit the timeframe of the dialogue to max. 30–40 min and to avoid the stress that might be caused by having to read and choose between different cards. We decided therefore to skip the third conversation round on societal issues. In addition we offered not cards but a booklet as input for the conversation: on each page they would see only one possible future use at the time, and on the ‘values page’ we offered all personal values at once asking them to underline the values they considered most important. After each page we would ask participants to share their thoughts. During the conversation we wrote down a summary of what participants said on a white board, thus helping them to keep track of the conversation or to relate their input to things that had been previously said.

### Data collection

In total we conducted seven focusgroups. Before we characterize the participants of our focusgroups as ‘patients’ or ‘non-patients’, it is however important to note that this distinction is not uncomplicated in this research. In fact, who counts as a ‘patient’ can shift as an effect of the development of a new diagnostic method, such as the detection of CAA biomarkers in brain fluids which promises to enable diagnostics in pre-symptomatic phases. Some people may be ‘patients’ according to such a diagnosis, while not experiencing complaints.

As we wanted to explore different perspectives to such a CAA-test, we therefore start to think about health and disease as extremes on a continuum. The participants in our focusgroups are positioned in different places on this continuum: we did two focusgroups with people who received the diagnosis of dementia and experienced moderate to severe complaints (four and nine participants respectively). Two focusgroups with people who had intracerebral hemorrhage due to CAA and whose lives were marked by the effects of it (three participants each). In addition we did one focusgroup with people from the HCHWA-D group, some of whom had previously taken a genetic test to find out whether they have the inheritable form of CAA that runs in their families (six participants). Some of the participants in this HCHWA-D group (but not all) experienced mild to moderate complaints. In addition, we did one focusgroup with elderly people who considered themselves healthy (seven participants), while some of them did experience mild, but undiagnosed, memory losses; and one focusgroup with partners of people with dementia (seven participants) who had experienced no dementia complaints themselves. Finally, we conducted two individual interviews with people who had had intracerebral hemorrhage due to CAA. In the end we had to exclude one of the interviews since we were unable to complete it with this interviewee. In total we based the analysis on conversations with 40 people.

Participants were recruited in different ways. Elderly people were recruited by means of an advertisement in three supermarkets located in different neighborhoods in Nijmegen, a city in the east of the Netherlands; people with dementia as well as partners were recruited via two nursing homes in the middle and east of the Netherlands. We included people who suffered cerebral hemorrhages who consented to participating in the clinical trial of our project and asked them whether they would be willing to participate in a focusgroup too. HCHWA-D patients were recruited via the HCHWA-D patient organization. We were careful to select men (21) and women (18) from different socio-economic backgrounds and levels of education. We did not attract people from different ethnicities and cultural backgrounds.

In the case of people with dementia we conducted the focusgroups in the nursing home, as this was a safe and well-known environment for them and would not cause stress. All other focusgroups were conducted either in a meeting room at a location that was convenient to the participants, in a nursing home, or at the university medical center. Two participants who had intracerebral hemorrhage were unable to read and were helped by the researcher (SvdB) during the focusgroup.

### Data analysis

**Fig. 1:**
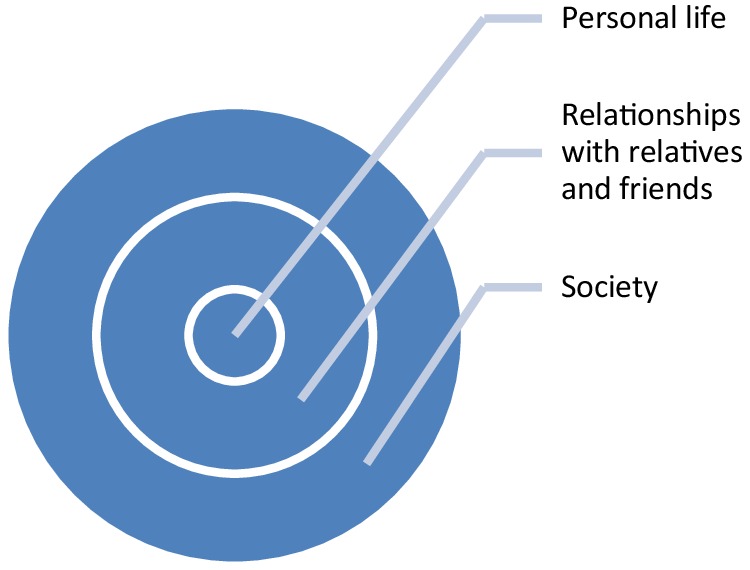
Three levels of reflection and evaluation about the value of CAA biomarkers

The focusgroups have been recorded and transcribed verbatim. As we were interested to explore the meanings and values attached to (presymptomatic) CAA detection, we conducted the analysis using a grounded theory approach, in which the codes, themes and codebook emerge from the data (Glaser and Strauss 1967; Lingard et al. 2008; Tong et al. 2007). At first we analyzed the transcripts of the groups of participants separately, as we thought this would give insight into how their diverse personal experiences with the disease (or lack thereof) would reveal different meanings and values related to CAA biomarker research. There was, however, a lot of overlap in the ways people understood and valued CAA detection. In the second round, we therefore chose to analyze all groups together and focus on commonalities and differences in the values and meanings that arise from them. In the first round we linked passages in the transcripts to codes, such as : “knowing is accepting”, “knowing makes less uncertain”, “knowing causes fear”, “caring for family”, “insecure social relationships”, “preparing for the future”, “preparing for death”, “contribute to the health of future generations”. Subsequently we ordered the codes under themes, which allowed to acquire insight in their ways of reflecting on the meaning and value of CAA biomarker research. We chose themes such as knowing the cause of complains, “adapting one’s life” and “timing of the diagnosis”. As we saw that participants in the various groups attached different meanings and values to the future use of CAA-biomarker detection in their own personal lives as opposed as how they would reflect on this in their relationships to family and friends, or with respect to society, we chose to order the presentation of the results accordingly. We presented these ways of reflecting graphically in Fig. 1.

In the following we will order our presentation of the results of our focusgroups and interview according to these three levels of understanding and valuing.

## Results

### Personal life

Respondents primarily reflected on the value of a diagnosis at a personal level. A diagnosis was considered important to know the cause of complaints and adapt future plans to the prospect of disease-development. This line of thinking was pursued when considering the value of the future possibility of a presymptomatic diagnosis.

#### Knowing the cause of complaints

Having a diagnosis is considered valuable to get more clarity and certainty about the cause of complains. This view, which we encounted in the various focus groups, is well captured in the following quote from a woman who explains the value of a diagnosis for her:

Well, yes, then you understand why you forget things or fail to understand.

(people with dementia)

Respondents furthermore appreciate biomarkers as a way to distinguish between different causes of complaints; such as, to separate between dementia and a burnout, or to distinguish the type of stroke, either an infarct (caused by a blood clot for which antithrombotic treatment should be given) and a cerebral hemorrhage (caused by rupture of blood vessel in which case antithrombotic treatment should be avoided).

… I would say that it can probably help to have something that indicates quicker that it is not a burnout. We took measures, all kind of measures to diminish stress … but the complaints didn’t go away, for it wasn’t a burnout. (partners)

… I had a major cerebral hemorrhage (..) The doctor immediately gave blood thinners. But afterwards we found out…in me the blood flow is too good. The blood thinners were dangerous for me… (intracerebral hemorrhage)

Values such as knowledge and control were considered important in this respect, as acquiring appropriate knowledge about the causes of complaints is considered important to tailor an appropriate response to the disease and to obtain some measure of control.

#### Adapting one’s life

Obtaining a diagnosis also marks the beginning of a process in which people have to adapt their life-plans and integrate the diagnosis into their self-perception and expectation for the future. This is often a process that is characterized by dealing with emotions such as anger and fear. The following quote provides an example:

…The diagnosis spoiled my life for a large part…My fear…My fear [is] that my life will end as my sister’s did. [the sister also had dementia] (people with dementia)

Values such as acceptance, finding joy in things one can still do, trust and (religious) faith, play a prominent role in how participants think they should deal with the diagnosis. But doing this is often considered difficult:

It [the diagnosis] is not something that makes you happy, but if you come as far as to accept and say ‘I’m going to try to make the best of it’ then you can give it a positive side. Well, that’s what I try at least. (people with dementia)

Some fear, yes yes…I think it is a kind of process. For me it lasted a year until I could give it a place [in my life]… I was very down for quite a while, but because of my trust and faith I came out of it. That is something I would like to give to everyone, but I can’t. It is something you can live with, I am personally convinced of that. Enjoying the day… I am not a special human being: everyone can do that. You just have to accept that you have something that won’t go away … (HCHWA-D)

#### Timing of the diagnosis

Participants predominantly reject presymptomatic diagnostics because (a) there is no appropriate therapeutic response available, and (b) it is not desirable to adapt one’s personal life prior to the experience of complaints. This is illustrated by the following quotes:

No. What can you do with it? You can do nothing with it. There is no cure… nothing… (people with dementia)

If you know early that you are developing CAA, you could organize your life around it… That seems undesirable, for it could mean that you close off your life before it is time… (partners)

Furthermore, some participants mention that obtaining a diagnosis presymptomatically might cause useless fear:

… how can you say you know what will happen? You can die of another disease… you are made scared of dementia and you die of cancer. Yes… that can happen, right? (people with dementia)

In the HCHWA-D focusgroup participants reflected extensively on the value of presymptomatic diagnoses, as they had experience with it in their own lives. The value of an open future played a prominent role in their reflection:

…it robs you of your careless attitude towards the future … and that should not be the case when you’re twenty. (HCHWA-D)

The HCHWA-D patients argued that one should not adapt one’s life-plans to the disease too early in life, because it can influence decisions about life such as forming relationships, having children, or choosing a profession. It is for this reason that participants advised their children not to do it too early. In their own lives, however, some participants considered it important to have the presymptomatic diagnosis, as it helped them cope with the stress they experienced living in a family with HCHWA-D.

I am of the category, I am happy I had myself tested… I was already restless because it is in the family. And in this way [with the test] you at least have the possibility to rule it out. … So I said: I can rule it out if I don’t have it, and if the message goes in the other direction than that is a pity. But I cannot say that it makes me more stressed now that I know. I could not live very well with the uncertainty... (HCHWA-D)

Participants in the HCHWA-D group would not promote a presymptomatic diagnosis for people who do not have CAA in their families. Even within their own group they observe that the choice to be tested is highly personal, and they see less reason to test members of the healthy population.

… we have the inheritable form and even in our community people relate to it [the testing] differently. Sometimes even in the same family some people know, and others don’t. It is a sensitive matter… So, it is questionable whether you should tell people who have nothing in their families … who have no bleedings [intracerebral hemorrhages] and who do not have any cognitive problems. I think that is quite something…I think you shouldn’t do it. (HCHWA-D)

### Relationships with relatives and friends

Our respondents made clear that obtaining a diagnosis marks the beginning of a process of change in relationships with relatives and friends. This comes forwards in their communication habits, the responsibilities they adopt towards each other and in their shared future planning and—for some—in their reflection about end of life decisions. Here we will focus on the last two aspects.

#### Adapting responsibilities

Once someone has dementia complaints, the division of tasks in the household often have to be revised. Participants in our focusgroups told many stories about how the adjustment of mutual responsibilities can go wrong, which is often blamed on a tendency to deny the diagnosis on the part of the patients:

He denied it [the disease] for a long time. It is only since a few months that he will tell people at our farm, the veterinarian for example: would you write that down for me, for I cannot remember things very well anymore. Now that is since three, four, five months, although we know for sure what is wrong with his memory since eight years! (partners)

Others told stories about a relative’s denial of the decline of their capacities:

… and I told him [father] that I would not allow him to take my children in the car anymore. It was dangerous, really…but he once took them without telling me. I was furious… and he would say: I know what I am doing… I know it is hard to accept that you can’t do things you used to do… But, boy, I was so afraid… in the end I only brought my children over when my mother was around. (HCHWA-D)

Patients, on the other hand, provide insight into their perspective to the matter, sometimes understanding very clearly that they are not able to live up to the expectations of their loved ones:

…I keep fighting until I can repair my relationship, for it is fragile right now as my wife thinks I have changed. I can understand that. I have become very passive… in everything really, you know. I lack initiative. I sit quietly in a chair at home and have nothing to do. I don’t mind that at all. And the whole room is full of dishes that need to be washed and my wife says: what do you think of that? Oh yes, that needs to be done…Nothing comes from me. I sit and I think it is OK. I don’t feel I miss anything either, you know. … Now sometimes she makes lists of things I should do that day. I don’t mind that. I do it all. (intracerebral hemorrhage)

Others tell that they think their relatives are too pushy to take over responsibilities, too patronizing, which is experienced as stressful:

…They walk in like… I have this and that for you… I have lived alone for many years and do everything myself. They took my car at a certain moment and, no…I’d rather have that they didn’t know I have dementia. (people with dementia)

Stories such as these reveal a struggle between family members to find a new balance in their mutual division of responsibilities. In some cases participants also tell stories about situations in which they experience this balance:

I used to cook quite well. Sometimes I cooked for many people. That was no problem for me. I just did it liked this [she makes a juggling movement]. But now I can’t do that anymore. Now my husband does the cooking. And I help. I cut something, for example…it is nice to stand in the kitchen together. (intracerebral hemorrhage)

#### Planning the future

Patients as well as partners and healthy people mention valuing a diagnosis in order to be able to organize the right professional and informal care for the future. Sometimes, patients anticipate needing care themselves:

We arrived at a point where we wanted to change our life, so we decided not to have a big garden, big house, and to move to a place not too far from our family. A little more centralized and we did that because there is indication that I have Alzheimer. (people with dementia)

Sometimes people anticipate needing care themselves, but also consider the needs of their relatives:

I think I will be all right in a nursing home and that my family should be able to live their own lives. … They didn’t choose this and my husband did not choose me knowing that I have this disease and if I myself would have known I had the disease then I probably would not have a relationship at all… I don’t know. (HCHWA-D)

Healthy elderly people and partners talk about providing care, but patients also often talk about themselves as people who care for their relatives; for example, by leaving them free to live their life, or trying to prepare everything as well as possible in case their capacities decline or they die:

I don’t know of course how this will continue…the development of my disease is unknown, for I don’t know when … when I will get a bleeding [intracerebral hemorrhage] again. But I want to leave things behind in a good way. … So I prepare everything. I organize my administration, so that my wife knows where she can find it …she will have enough on her mind… (intracerebral hemorrhage)

Organizing care facilities for the diseased in such a way that family members will be able to continue living as good as possible is a recurrent theme in all of the focusgroups. Obtaining a diagnosis is important for it marks the beginning of preparatory activities to care well for each other. Obtaining a diagnosis too early is not appreciated in this respect, for it would urge people to take preparatory measures at a moment when this is not yet necessary.

#### Timing of end of life decisions

Having a diagnosis early (presymptomatically), or being able to monitor the stage of development of the disease, is appreciated by some respondents, as it informs decisions about the best time to ask for euthanasia. As euthanasia is an accepted practice in the Netherlands, some patients reflected on the difficulty of picking the right time for it. This can be problematic for people with declining cognitive capacities, for the Dutch law requires patients who request euthanasia to be able to make an autonomous decision. The participant we interviewed anticipated that a presymptomatic diagnosis, or a tool that could monitor the progression of the disease prior to experiencing its symptoms, could help to make the decision at the right time:

I do not want to come so far as I don’t…don’t know what I do anymore… that my wife has to take me to a nursing home, for example. I had a beautiful life and I don’t want… don’t want to end up all strange. I talked about it [euthanasia] with my family doctor… a few times already, but she doesn’t want…yes, what is the right time? I want to prevent that I deteriorate and can’t ask for it anymore. So if there would be a way to see how far I am in the disease, this would be helpful for that, yes. (intracerebral hemorrhage, interview)

In the HCHWA-D focusgroup choosing the right time to graciously close off life was also a recurrent theme, although euthanasia was not mentioned. Some of the participants had themselves tested and know they have inherited the genetic variant that causes the disease. During the focusgroups they expressed that they go through periods of extreme stress when relatives suddenly die or rapidly develop cognitive deterioration. Stress causes sometimes complaints very similar to dementia, which causes even more worries. It is for this reason that they would value having a more objective instrument that can tell them the cause of their complaints, like a monitoring tool that can tell them how far the disease has progressed and allows them to take measures in time.

… now it is common in the hospital to say ‘you have the gene but we cannot do anything with it, so please come back some time.’ Now I think that for some people, that is fine, but if you…I experienced that I started misunderstanding things…like I was already in the middle of the disease process… while I was actually suffering a burn-out… and then you don’t know how to understand it. Then I found it very important to be monitored in order to know: should I be concerned, should I take measures? … I am a psychiatrist in a practice… If I know that I am far in the process, then I would stop. (HCHWA-D)

In the lives of people with HCHWA-D having a way of monitoring their disease progression allows them to act responsibly and not engage in work that they can no longer do. Another reason for patients with HCHWA-D to want to use biomarker information for monitoring is that this would allow medical specialists to tell them when their condition is deteriorating, rather than burdening their own family with it.

Imagine you are more affected than you yourself think… It can happen that you don’t see things yourself. I, for example, told my husband that if I start doing strange things, I want you to tell me. You also see it in the patient association: some people—if I may say so—have a high opinion of themselves. Then it is valuable to have something objective … In a relationship you spare each other … For that reason I would value having an objective measure that can tell me: how am I doing? (HCHWA-D)

Participants in all other focusgroups were however not so much interested in a monitoring function of the biomarker-detection method. To some it was considered a frightening option to be able to follow your own degeneration, while not being able to stop it. The chance to make informed choices about when to stop working or start to close off life does not figure in all participant’s minds, and some consider it a frightening combination with euthanasia as they think presymptomatic diagnostics might lead to even less societal acceptance of dementia and an implicit societal push to choose euthanasia.

Woman: I think you will get more euthanasia. [when you get a diagnosis early. SvdB]

Researcher: more euthanasia?

Woman: Yes, more euthanasia.

Researcher: Why more euthanasia?

Woman: The word dementia means clean up and put in the corner. (people with dementia)

I would probably choose euthanasia myself… I don’t know. But I also think… well…if you live in a country where euthanasia is allowed, and you give people information that they have dementia early on… well what can you do with that information? I’m afraid people will probably think they have to choose euthanasia, as if it is the only reason… why they get this information. I think that would be wrong. People should not feel they have to do something like that. (partners)

### Societal aspects

Participants also reflected on the value of biomarker diagnostics for society. The issue they chose to talk about most was the issue concerning what society should primarily invest in: (1) in the provision of good care for people; or (2) into the development of medication. Sometimes the respondents looked at this issue as if it asked for an evaluation of science itself, which they valued for the sake of itself:

I am in favour of science. Period. Yes, I am like that. I am a curious person. (Interview patient with intracerebral hemorrhage)

But many participants reflected more specifically on the kinds of benefits they expected from scientific research, and how they would be able to use that to care for people. They consider it beneficial, for example, to have more information about the disease, including its various complaints and stages. This allows people to prepare, as they know better what to expect:

(..) it [science] allows to observe the development of the disease …This helps to understand the disease and tell people about it when they get it. (healthy elderly people)

Or some actually looked at the value of follow-up research into the effectiveness of medication, which makes patients and their care-givers less helpless. For this purpose, some consider it valuable to do research on people, even if they do not yet have symptoms. That may help to prevent irreversible damage from occurring:

I think that if you do research in people who do not have symptoms yet, who stand at the beginning [of the disease], then maybe it is possible to develop medicine… Because now…when someone has it…it is actually already too late. (partners)

A medicine, that’s what we hope for. Then you can precede such a bleeding [an intracerebral hemorrhage] and prevent damage. (intracerebral hemorrhage)

For the HCHWA-D group, the focus on research is especially important, as they contribute in that way to the health of future patients, including their own children. Participants in this group that is marked by a family history of disease, find it important to take part in research to be able to care for the future health of their children:

Research is very important and you can perceive in our group that people are willing to do a lot to realize that. Because we all want research to go fast for our offspring, so yes, research is important in our group … for our children, our grandchildren. (HCHWA-D)

All of these remarks reveal that participants think that more knowledge and medication will help to care better for future patients. But not all participants agree with this. Some participants expressed a basic feeling of distrust towards science and scientists:

For whom are scientists working? I don’t know, I am probably seeing things too negative. But it is their work and they can obtain high prestige, they can earn money. Are they concerned with us, with patients and their families? I do not think so. (partners)

Other participants perceived a distinction between the search for cure and care. They thought that more money should be spent on caring for the people with dementia in society, rather than spending it on finding innovative medication. They were not convinced that knowledge would eventually lead to more care, and expect more from general supportive activities for patients.

I am quite skeptic… searching for medication… I also see good results when there is good support and accompaniment … then there is a lot less risk ... I think there should be much more attention for what you can do with good care. Society does not sufficiently recognize this. (healthy elderly people)

I know that it can have an enormous impact on your life when you become the guardian of your father or mother. We became ill ourselves, my brother and I. I think this is not good. It is not good that in the Netherlands we now burden informal care-givers even more. I think, come on boys in the government, please work behind that front door for a while and then you will talk differently. It is really important to give care-givers some air, to let them live a little bit… (healthy elderly people)

Pondering on whether societal money should be spent on science or on better caring facilities, especially the groups with healthy elderly people and the partners reflected on the appropriate attitude that should be adopted and fostered towards diseases of old age in society:

… I think, how should I say this, yes you can spend a lot of money to get to know everything, but the question is whether … that is worthwhile… You have to accept that there are things in life that happen and that you cannot influence. Let’s just put it like that. (partners)

In all of these answers, care seems to play a prominent role. Advancing knowledge is valued by some participants in our focusgroups as a way to strengthen capacities to care for patients. But other participants value acceptance of the limitations of life and think this is a lesson to learn for society at large. They think society should not understand the degeneration of capacities towards the end of life as signs of disease that can be cured, but as part of the natural life cycle. Seeing it like this would make caring for people with dementia a more natural part of everyday life, and would help to see the need to develop caring capacities. If dementia is seen as an anomaly that demands to be ‘fixed’ with medicine, this does not inspire to train caring capacities which are needed when people grow old.

## Discussion

Given the described results, we conclude that (future) patients do not only—and not primarily—value biomarker detection in relation to the possibility of future treatments for CAA related diseases. In the absence of treatment, (presymptomatic) CAA detection or monitoring is valued to the extent that it helps people to care for themselves, for family and friends or for society. This is the case in circumstances in which it (a) explains experienced complaints, (b) allows to start a process of psychological acceptance and social adaptation to the expected degeneration, (c) contributes to dealing with anxieties (with respect to inheritable versions of dementia), (d) informs adequately about when to start preparing for the end of life, (d) informs the planning of a request for euthanasia, or (e) allows society to deal with a growing number of dementia patients. CAA detection or monitoring is valued only when it enables people to do something with the results in their (self) care; it is not valued when the recipients of disease information feel unable to do anything with that information in their care, or if they do not feel cared for. In this case information about disease can be considered ‘premature’ and ‘harmful’ as they may feel the disease obliges them to anticipate the end of their lives while they do not know how.

Our results offer a contribution to the scholarly debate about what the right approach is to the expected rising amount of dementia patients with which this article started. We described in the introduction of this article that social scientists and ethicists often defend a care response to the growing number of people suffering from cognitive decline, which they contrast with the cure response that they ascribe to biomedical researchers investigating the biomarkers responsible to the development of CAA. The results of our focusgroups, however, reveal that participants do not always see care and cure strategies as rivals. Instead of looking at biomarker research as a stepping stone towards research into the effectiveness of therapy, participants in our focusgroups tended to evaluate future use of biomarkers with respect to whether and how they expect it to enhance their capacity to (self) care: they look at whether it fosters their capacity to deal with experienced complaints, with emotions, with (unstable) relationships with others and determine whether and how it can help them to organize support when their capacities will decline or support them to make decisions about how to end life graciously. In other words, they value the future prospects of biomarker research with respect to how it helps them to care for themselves and others, even if treatment is (and stays) unavailable.

Our study therefore suggests that end-users have tendency to evaluate future CAA detection and monitoring from a care perspective. But care is not contrasted with cure. Care is understood very broadly, in the inclusive definition provided by care ethicists Joan Tronto and Berenice Fischer as ‘(..) a species of activity that includes everything we do to maintain, contain, and repair our ‘world’ so that we can live in it as well as possible (..)’ (Tronto 1993, p. 103). Knowing that CAA is at present not an aspect of their world which can be repaired, as there is no ‘technological fix’ available at present, and it may not become available in the future, participants in our focusgroups evaluate future uses of CAA biomarker detection for its imagined capacities to empower them to deal with CAA with resilience in their personal lives. While their personal lives and relationships differ as well as their personal history with CAA -which is visible in their varied evaluations- they have in common that they evaluate CAA diagnostics with respect to how it can support them to care for themselves and their family and friends. If treatment would become available in the future, this will be evaluated too for its influence on their capacity to care. Care and cure are therefore not opposed; rather, cure is one of the elements that contributes to care. If it would be possible to develop treatment, it would likely be evaluated according to its capacity to diminish or postpone complaints of disease and help people to maintain or repair their worlds.

This care perspective is also recognizable when they consider the societal value of this type of research. While participants disagree as to whether it is possible, or valuable, to do research into a cure, they have in common that they use a care-vocabulary in their evaluation. Some participants consider finding a cure a way to improve care for future generations of patients, others consider it more caring to improve supportive facilities in order to make sure that society can maintain a good quality of life for the growing amount of people suffering from cognitive decline. While they are in disagreement about the value of finding a treatment, they do not oppose care to cure in their societal evaluations care.

While it is innovative to explore the meanings and values attached to innovative CAA detection in brain fluids, the results of our study also has a lot in common with other qualitative studies exploring how Alzheimer patients and their informal caregivers experience diagnoses offered by current diagnostic methods (Bunn et al. 2012; Spreadbury and Kipps 2017; Robinson et al. 2011; Greenwood and Smith 2016). These studies also describe that respondents think that a diagnosis (a) helps to understand the cause of complaints, (b) impacts on identity, roles and relationships, (c) contributes to a better understanding between patients and their relatives, (d) allows caregivers to adjust to increased responsibilities and become the main decision-maker, (e) to organize care and support in the present and towards the future, and (f) develop strategies to support or minimize the impact of dementia. As these studies focused on personal experiences with current Alzheimer diagnostics, they did not include a reflection on future ways to diagnose the disease, nor on the value of changing diagnostics. Furthermore, they did not look at the connection between these elements in a care approach. This is what our study adds.

The care perspective we identified in the responses of our respondents suggests, in our view, an interesting new way to look at the value of CAA biomarker research. This research bears not only the promise of finding a ‘technological fix’ for dementia, as authors such as Jongsma and Sand suggest in their article in *Medicine Healthcare and Philosophy*. The (presymptomatic) detection or monitoring of CAA biomarkers can also be valued for its capacity to foster capacities to (self) care. Research into CAA detection or monitoring should therefore not solely be understood and valued as a stepping stone towards follow-up research into innovative medicine. While medicine could contribute to (self)care, it is not sure whether and when it will be realized. This will depend on extensive research during the coming decades. In the meanwhile, it is important to anticipate the benefits and harms that will result if the promise of finding a treatment will not materialize. Therefore, it should also be assessed carefully how the availability of a CAA detection and monitoring method can foster capacities of patients and their care-givers to care for themselves and each other, and how it is able to undermine that capacity, in the absence of treatment. We think our study provides input to make such an assessment, which depends on the contribution it is able to offer to ‘good (self) care’ in different circumstances and to different people.

Our study suggests that information about disease is considered ‘harmful’ or ‘premature’ when recipients feel unable to act on that information in their (self-)care. In these situations recipients of information about CAA building up in their brain fluids may think that this obliges them to anticipate the end of their lives, while they are unsure who or what will help them to do that. This is our reason to think that it would be good to study whether and how CAA detection can be embedded fruitfully in palliative care. If palliative care is understood in a broad sense, such as the World Health Organization recommends, it starts when patients receive a diagnosis of an incurable disease and it may last for years. Furthermore, palliative care adopts a broad perspective to care, which takes into account the entire person of the patient, including physical, psychosocial and spiritual needs. Palliative care could therefore help to prevent the harm caused by a ‘premature’ diagnosis, by offering support to deal with this knowledge and integrate it in one’s personal life, as well as relationships with others.

Palliative care could support patients to deal with early diagnoses based on biomarkers, but biomarker diagnostics can probably also foster palliative care. It is a recognized problem that physicians often start the conversation about palliative care too late, when patients are no longer able to express their wishes and needs. Described obstacles to shaping a timely advance care plan are that physicians (a) find it difficult to cohere palliative care with their curative role as physicians, (b) are afraid to start talking with patients about palliative care as they fear patients or their families will be unwilling to talk about it, or (c) fail to recognize that patients are already in the palliative phase (Oliver et al. 2015; Kupeli et al. 2016; Voltz 2016). If physicians and patients don’t shape an advance care plan in time, patients will fail to receive support and care tailored to their values and wishes as they will not have had the chance to communicate about it. It would be worthwhile to investigate whether CAA biomarker research can contribute to solving this problem, and start a timely conversation about palliative care. It would respond to a well described need in palliative care to make an advance care plan in time and it coheres with the perceived value of biomarker research as a way to contribute to self-care, care for relatives, care for their own end of life, and care for future generations that we encountered in our study.

Of course, the connection between CAA diagnostics and palliative care also raises difficulties, as people who receive information about CAA building up in their brain fluid may not be ready to consider themselves palliative patients and resist to talk about palliative care. This is a problem that requires more attention in future research, for when CAA diagnostics becomes available it can be expected that more people will receive their diagnosis earlier. Furthermore, in some contexts that allow euthanasia—such as the Netherlands or Belgium—early diagnoses can raise particular sensitive issues. As our study suggests, (future) patients and their care-givers consider the value of (presymptomatic) CAA detection and monitoring also in relation to euthanasia. Obtaining an early diagnosis can be experienced as a help to ask for euthanasia timely, before the degeneration of the brain prevents making an autonomous choice (which is a prerequisite of euthanasia). For others, however, detecting CAA early may produce a feeling in some patients that they are somehow expected to choose euthanasia by their social environment. It would be advisable, in our view, if future research and ethical debate develops an appropriate response to these feelings, by means of integration of CAA diagnostics in care that is tailored to the person of the patient. Person-centered care is the primary goal of palliative care. Exploring how to integrate CAA detection and monitoring in a palliative care trajectory, would, in our view, be therefore a promising direction for future research, which attends to (new) needs that early diagnostics can generate, and fits with the care-language that (future) patients already adopt.

Of course it needs to be noted at the very end that our study has limitations too. We did not include participants from different cultural backgrounds, which limits the input that we collected. This is definitely an omission that needs attention in further research in the future. Furthermore, we shaped the cards that enhanced reflection and discussion in the focusgroups based on interviews. These interviews were carried out with a limited group of researchers and patients, which may have led to limited the amount of topics that our cards included. Even though participants in the focusgroups were free to start talking about other topics, the choice of topics on the cards may have constrained the range of topics that was discussed. What is missing, for example, was a discussion of scenarios about possible misuse of information after a CAA biomarker test, or questions concerning how patients’ data should be handled. Future research in this area could include these topics as well.
